# Validity of a stroke severity index for administrative claims data research: a retrospective cohort study

**DOI:** 10.1186/s12913-016-1769-8

**Published:** 2016-09-22

**Authors:** Sheng-Feng Sung, Cheng-Yang Hsieh, Huey-Juan Lin, Yu-Wei Chen, Chih-Hung Chen, Yea-Huei Kao Yang, Ya-Han Hu

**Affiliations:** 1 Division of Neurology, Department of Internal Medicine, Ditmanson Medical Foundation Chiayi Christian Hospital, 539 Zhongxiao Road, East District, Chiayi City, 60002 Taiwan; 2 Department of Neurology, Tainan Sin Lau Hospital, 57, Section 1, Dongmen Road, East District, Tainan, 70142 Taiwan; 3 Department of Neurology, Chi Mei Medical Center, 901 Zhonghua Road, Yongkang District, Tainan, 710 Taiwan; 4 Department of Neurology, Landseed Hospital, 77 Guangtai Road, Pingjhen District, Taoyuan Taiwan; 5 Department of Neurology, National Taiwan University Hospital, 7 Zhongshan South Road, Zhongzheng District, Taipei, 10002 Taiwan; 6 Department of Neurology, National Cheng Kung University Hospital, College of Medicine, National Cheng Kung University, 1 University Road, East District, Tainan, 701 Taiwan; 7 School of Pharmacy, Institute of Clinical Pharmacy and Pharmaceutical Sciences, College of Medicine, National Cheng Kung University, 1 University Road, East District, Tainan, 701 Taiwan; 8 Department of Information Management and Institute of Healthcare Information Management, National Chung Cheng University, 168 University Road, Min-Hsiung, Chiayi County 62102 Taiwan

**Keywords:** Acute ischemic stroke, Claims data, Disease severity, National Health Insurance Research Database, Outcomes research

## Abstract

**Background:**

Ascertaining stroke severity in claims data-based studies is difficult because clinical information is unavailable. We assessed the predictive validity of a claims-based stroke severity index (SSI) and determined whether it improves case-mix adjustment.

**Methods:**

We analyzed patients with acute ischemic stroke (AIS) from hospital-based stroke registries linked with a nationwide claims database. We estimated the SSI according to patient claims data. Actual stroke severity measured with the National Institutes of Health Stroke Scale (NIHSS) and functional outcomes measured with the modified Rankin Scale (mRS) were retrieved from stroke registries. Predictive validity was tested by correlating SSI with mRS. Logistic regression models were used to predict mortality.

**Results:**

The SSI correlated with mRS at 3 months (Spearman rho = 0.578; 95 % confidence interval [CI], 0.556–0.600), 6 months (rho = 0.551; 95 % CI, 0.528–0.574), and 1 year (rho = 0.532; 95 % CI 0.504–0.560). Mortality models with the SSI demonstrated superior discrimination to those without. The AUCs of models including the SSI and models with the NIHSS did not differ significantly.

**Conclusions:**

The SSI correlated with functional outcomes after AIS and improved the case-mix adjustment of mortality models. It can act as a valid proxy for stroke severity in claims data-based studies.

**Electronic supplementary material:**

The online version of this article (doi:10.1186/s12913-016-1769-8) contains supplementary material, which is available to authorized users.

## Background

With the advent of information technology, large amounts of health care utilization data are collected routinely for reimbursement purposes and the administration of health services. These administrative claims data offer several potential advantages to researchers, including large sample sizes, representativeness, being of large quantity and serial longitudinal, comparatively low cost, and relative efficiency [1]. Therefore, in addition to clinical epidemiological research [2], administrative claims data are appropriate for examining outcomes, performing pharmaco-economic analysis, and monitoring the quality of clinical care [3, 4].

However, because of insufficient clinical information, studies using administrative claims data have shared a crucial shortcoming, namely, a lack of adjustment for disease severity. This limitation is particularly relevant for stroke outcomes research because the heterogeneity of stroke syndromes causes stroke severity to vary greatly among patients. For example, studies based on administrative claims data that have investigated mortality and readmission rates in stroke patients have generally been limited by inadequate adjustment for stroke severity [5, 6]. In addition, claims data-based risk models for predicting mortality and readmission might be prone to mischaracterizing the quality of stroke care delivered by hospitals, because stroke severity is not included in the models [7]. Therefore, it has been advocated that more research be done to ascertain stroke severity using administrative billing codes or electronic health records [8].

Administrative claims data reflect routine clinical practice and could be analyzed as a set of proxies that indirectly represent the health status of patients [9]. In response to the pressing need for a better ascertainment of stroke severity using administrative claims data, we previously developed several models to derive a stroke severity index (SSI) that reflects the stroke severity of patients hospitalized for acute ischemic stroke (AIS), using only the information generally available in claims data [10]. Before the SSI can be applied to measure stroke severity in claims-based research, its criterion-related validity should be examined. Criterion-related validity is the extent to which the score from a new measurement instrument correlates with that of other measures evaluating the same or a very similar construct [11]. We have assessed the concurrent validity, a type of criterion-related validity, of the SSI by demonstrating its close correlation with the actual severity of neurological deficit at admission as assessed using the National Institutes of Health Stroke Scale (NIHSS) [10], the current gold standard to measure stroke severity.

In the current study, we aimed to evaluate the predictive validity, another type of criterion-related validity, of the SSI by examining the extent to which the SSI predicts future functional outcomes with a cohort of stroke patients by cross-linking hospital-based registry databases with a nationwide claims database. We also investigated whether the SSI has improved the case-mix adjustment in claims data-based outcomes research by examining the magnitude of improvement in model performance when the SSI was added to models that attempted to predict mortality in patients with AIS.

## Methods

### Study population

We identified adult patients with AIS in stroke registries from the 1300-bed Chi Mei Medical Center and the regional 600-bed Landseed Hospital in Taiwan. Patients admitted to the two hospitals between August 2006 and December 2010 due to AIS were included. Patients with in-hospital stroke were excluded. The study hospitals prospectively registered all stroke patients admitted within 10 days of symptom onset conforming to the design of the nationwide Taiwan Stroke Registry [12]. Ischemic stroke was defined as an acute onset of neurologic deficits persisting longer than 24 h with no hemorrhage visible in the first brain computed tomography or with acute corresponding ischemic lesion(s) on diffusion weighted magnetic resonance imaging. Stroke severity was determined using the NIHSS at admission. The NIHSS is a 15-item neurologic examination scale designed to assess neurological deficits in stroke patients. The total NIHSS scores range from 0 to 42, with higher values representing greater stroke severity.

**Fig. 1:**
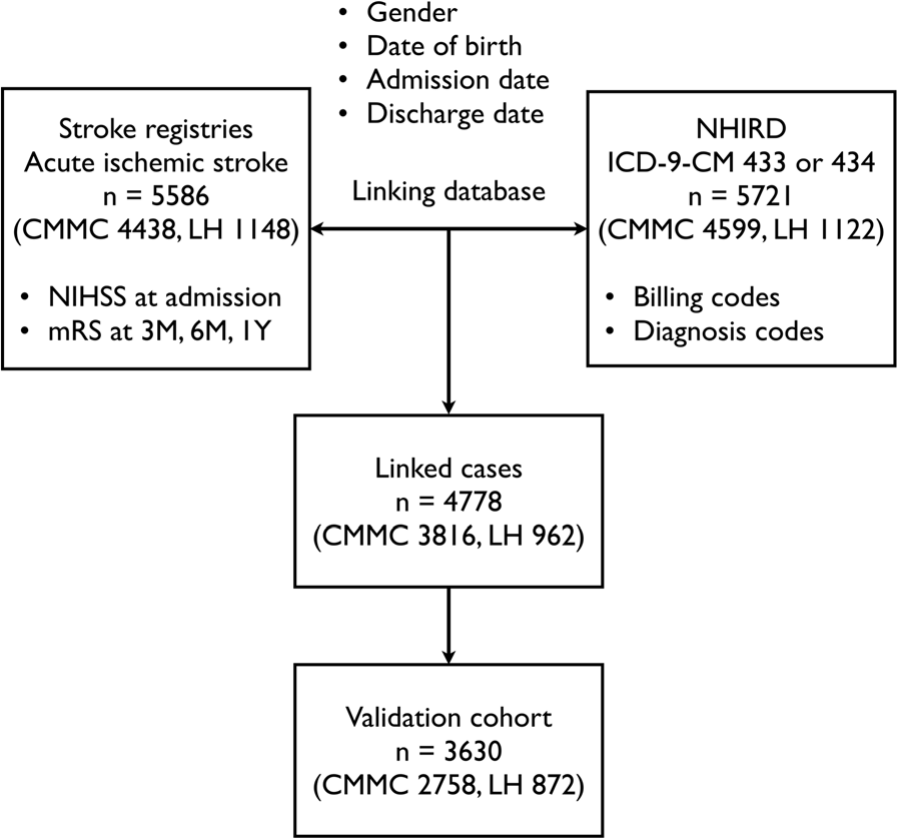
Flow diagram of study procedure. Abbreviations: CMMC, Chi Mei Medical Center; ICD-9-CM, International Classification of Diseases, Ninth Revision, Clinical Modification; LH, Landseed Hospital; mRS, modified Rankin scale; NHIRD, National Health Insurance Research Database; NIHSS, National Institutes of Health Stroke Scale

The functional status of patients who consented to follow-up was evaluated with the modified Rankin Scale (mRS) at 3 months, 6 months, and 1 year after stroke, during an in-person assessment or by telephone interview. The mRS is a commonly used tool for measuring the degree of disability of stroke patients. It is a six-point scale with 0 for no symptoms, higher scores for increasing disability, and 6 for death. Although a structured interview format has been developed to improve the assessment of the mRS [13], the mRS was determined based on the Chinese translation of the mRS criteria according to the Taiwan Stroke Registry operation manual [12]. We dichotomized mRS at ≤ 2 versus > 2 in accordance with previous stroke trials [14]. An mRS score of ≤ 2 indicates a good functional outcome (slight or no disability with preserved ability to look after own affairs without assistance). An mRS score of >2 means a poor functional outcome (dependence in daily activities or death). To assure patient anonymity, the data collected were limited to gender, birth date, admission date, discharge date, admission NIHSS score, and follow-up mRS scores from the registry databases (Fig. 1). Both the Chi Mei Medical Center Institutional Review Board and the Landseed Hospital Institutional Review Board approved the study protocol. Because the present study involved analysis of secondary data and all patient data were deidentified, a signed informed consent to participate in the study was determined unnecessary.

### National Health Insurance Research Database linkage

The National Health Insurance (NHI) is a mandatory, single-payer enrollment healthcare program that covers nearly the entire population of Taiwan. The program provides universal coverage for prescription medications, inpatient care, and ambulatory care. The National Health Insurance Research Database (NHIRD) comprises medical care claims data on NHI enrollees and is maintained and released for research by the National Health Research Institutes of Taiwan.

To identify the inpatient population of patients with AIS in the NHIRD, we extracted data on all patients hospitalized between August 2006 and December 2010 for ischemic stroke (International Classification of Diseases, Ninth Revision, Clinical Modification [ICD-9-CM] diagnosis code 433.x or 434.x) as their primary discharge diagnosis [15] from an NHIRD data set. We then linked registry data with the NHIRD according to 4 nonunique patient characteristics: gender, date of birth, admission date, and discharge date (Fig. 1) [15]. Because errors in coding or entering data in administrative claims data might be present, failure of linkage is inevitable [16]. Patients who were successfully linked and had follow-up mRS scores comprised the validation cohort. The linked hospitalization record in the NHIRD was defined as the index hospitalization.

For each case in the validation cohort, we obtained all the diagnosis codes from the index hospitalization as well as inpatient and outpatient claims within the 1-year look-back period before the index hospitalization using all the available nationwide claims records (Fig. 1). Patients were identified as having a comorbid condition if its corresponding ICD-9-CM codes appeared in at least one inpatient claim or three outpatient claims during the 1-year look-back period [17]. We estimated the modified Charlson comorbidity index (CCI) according to the ICD-9-CM diagnosis codes [18]. In addition, we retrieved reimbursement data for medications, laboratory tests, imaging studies, procedures, and clinical services from the index hospitalization, which were used to estimate the SSI for each patient.

### Stroke severity index

We have previously developed several SSI models using various regression methods, including *k*-nearest neighbor regression, regression tree, and multiple linear regression, based on the data from 3577 patients with AIS in a single hospital [10]. This index predicts the neurological deficit severity of patients hospitalized for AIS according to seven predictors (Table 1). These predictors essentially reflect the care given to stroke patients and management of stroke-related complications, which generally correlate with stroke severity [19, 20]. For example, among stroke patients, frequent airway suctioning is generally needed for those with difficulty in handling their own secretions, the placement of a nasogastric tube for feeding is usually required for those with dysphagia, bacterial sensitivity tests are often performed for those with severe stroke and therefore prone to infections, and mannitol osmotherapy is frequently prescribed for those with brain edema [19, 20].

**Table 1:** Multiple linear regression model for the stroke severity index [10]

Predictor	Coefficient
Airway suctioning	3.5083
Bacterial sensitivity test	1.3642
General ward stay	-5.5761
ICU stay	4.1770
Nasogastric intubation	4.5809
Osmotherapy (mannitol or glycerol)	2.1448
Urinary catheterization	1.6569
Constant	9.6804

*ICU* intensive care unit

An online tool (http://hdmlab.twbbs.org:508/SSI/hdmlab/ssi2.jsp) accompanying our previous study is available for estimation of the SSI using three kinds of regression methods. In the present study, we used the multiple linear regression model because it is simple to implement and to disseminate to other researchers. We determined the presence of each predictor according to the administrative billing codes (see Additional file 1: Table S1) listed in each patient’s claims from the index hospitalization. If a patient was transferred to another hospital during the index hospitalization, the claims records from the second hospitalization were ignored. The SSI was obtained by using the regression coefficients estimated from a multiple linear regression equation in our previous study (Table 1) [10].

### Statistical analysis

Continuous variables were summarized with mean (standard deviation) or median (interquartile range), and categorical variables with frequency and percentages. Two-tailed *P* values of < 0.05 were considered significant. To summarize the relationships between stroke severity and outcomes after stroke, the NIHSS was categorized as mild (≤5), moderate (> 5 to ≤ 13), or severe (> 13) stroke in accordance with a prior study [21], in which the NIHSS was used to predict stroke outcome. Because the SSI can be converted to the NIHSS as follows: NIHSS = 1.1722 × SSI − 0.7533 (see Additional file 1: Table S2), we categorized patients as having mild (SSI ≤ 5), moderate (SSI > 5 to ≤ 12), or severe (SSI > 12) stroke in accordance with the study mentioned above. Chi-squared tests for trend were used to evaluate trends across the categories of stroke severity.

To assess the predictive validity of the SSI (the extent to which the SSI predicts future functional outcomes), we examined the Spearman rank correlations between the SSI and the mRS at 3 months, 6 months, and 1 year. The correlations between the NIHSS and the mRS were also estimated. We further tested whether the correlations between the SSI and the mRS and those between the NIHSS and the mRS were equal [22].

To investigate the performance of models in predicting mortality at 3 months, 6 months, and 1 year after stroke, we fitted separate logistic regression models using age, sex, vascular risk factors (hypertension, diabetes mellitus, hyperlipidemia, prior stroke, atrial fibrillation, coronary heart disease, chronic kidney disease), and modified CCI as covariates with or without the SSI. The modified CCI was dichotomized into low comorbidity (0 or 1) and high comorbidity (≥ 2) for analysis [18]. The SSI was entered as a continuous variable. For comparison, additional logistic regression procedures were performed using age, sex, vascular risk factors, modified CCI, and the NIHSS as covariates. The NIHSS was also treated as a continuous variable. Model discrimination was assessed and compared using the area under the receiver operating characteristic curve (AUC) as recommended by DeLong [23]. We used integrated discrimination improvement (IDI) to evaluate the improvement of predictive ability after adding either the SSI or the NIHSS to the models [24]. A higher IDI indicates greater risk discrimination and improved classification. We assessed the model calibration by the Hosmer–Lemeshow goodness-of-fit test. All the statistical analyses were performed using Stata 13.1 (StataCorp, College Station, Texas).

## Results

In total, 4438 (Chi Mei Medical Center) and 1148 (Landseed Hospital) adult patients hospitalized for AIS were identified from the stroke registries of the two study hospitals. After linkage with the NHIRD, 3816 and 962 patients, respectively, were successfully linked (Fig. 1). Overall, the rate of successful linkage was 85.5 %. Compared with the unlinked patients, the NHIRD-linked patients were slightly younger and had somewhat lower NIHSS (see Additional file 1: Table S3). Among the 4778 linked patients, follow-up mRS scores were available for 3630 patients at 3 months after stroke, 3545 at 6 months, and 2478 at 1 year. Patients with follow-up mRS were marginally older and had slightly lower NIHSS than those without (see Additional file 1: Table S4). Table 2 lists the characteristics of the validation cohort. The mortality rates were 4.7 % at 3 months, 7.0 % at 6 months, and 14.2 % at 1 year, which were comparable with those (30-day mortality 4.2 to 4.3 %; 1-year mortality 13.9 %) found in previous nationwide studies [25, 26].

**Table 2:** Characteristics of the validation cohort

Characteristics	*n* = 3630
Age, mean (SD)	67.6 (12.7)
Female	1474 (40.6)
LOS, median (IQR)	5 (4–9)
Modified CCI	
0 or 1	2512 (69.2)
≥ 2	1118 (30.8)
Vascular risk factors	
Hypertension	2729 (75.2)
Diabetes mellitus	1658 (45.7)
Hyperlipidemia	1445 (39.8)
Prior stroke	689 (19.0)
Atrial fibrillation	360 (9.9)
Coronary heart disease	198 (5.5)
Chronic kidney disease	103 (2.8)
Stroke severity	
NIHSS, mean (SD)	7.1 (7.8)
SSI, mean (SD)	6.8 (4.8)
Components of SSI	
Airway suctioning	542 (14.9)
Bacterial sensitivity test	553 (15.2)
General ward stay	3602 (99.2)
ICU stay	387 (10.7)
Nasogastric intubation	802 (22.1)
Osmotherapy (mannitol or glycerol)	192 (5.3)
Urinary catheterization	742 (20.4)

Data are numbers (percentage) unless specified otherwise

*CCI* Charlson comorbidity index, *ICU* intensive care unit, *IQR* interquartile range, *LOS* length of stay, *NIHSS* National Institutes of Health Stroke Scale, *SD* standard deviation, *SSI* stroke severity index

**Fig. 2:**
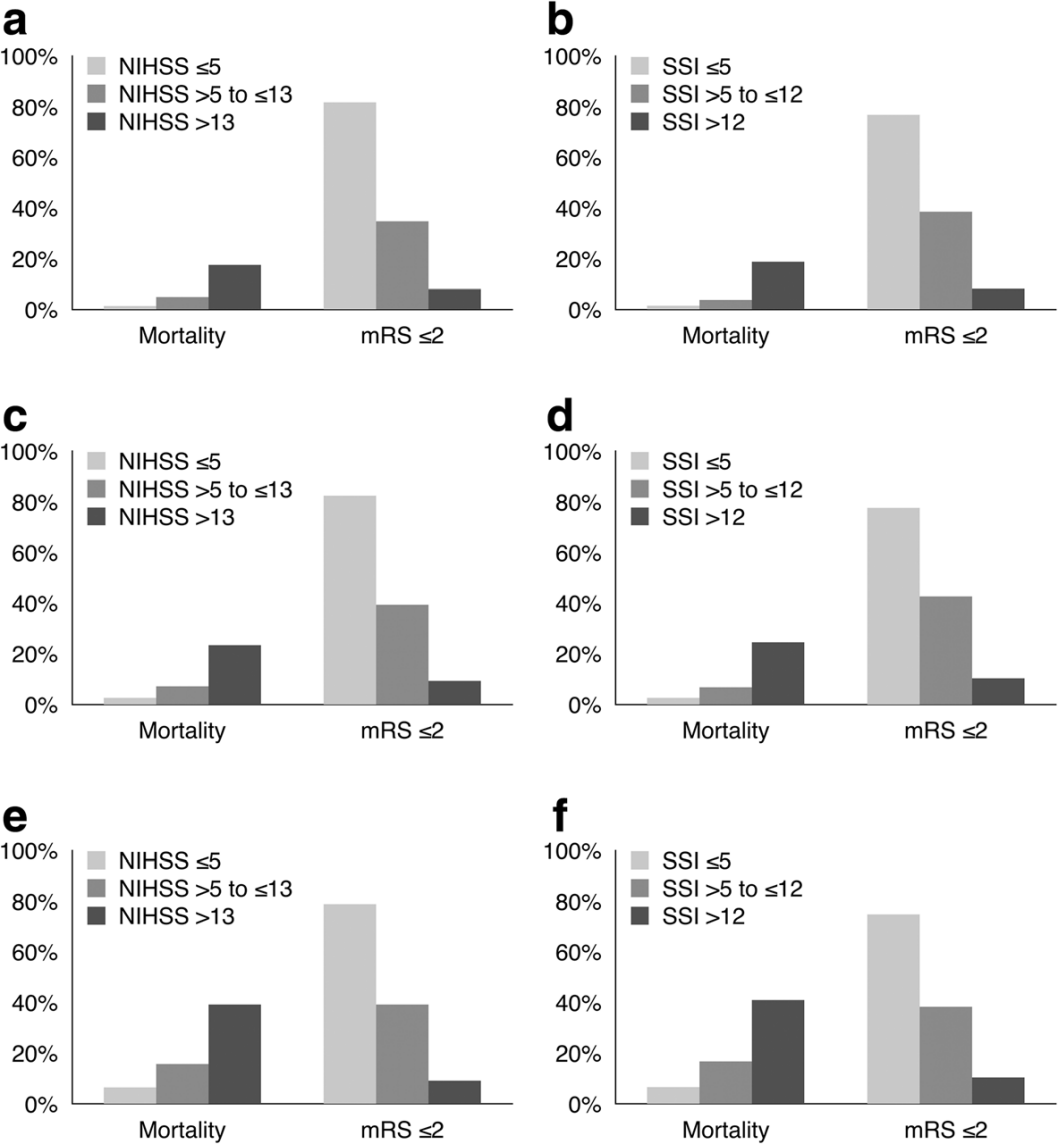
Mortality and proportions of good functional outcomes stratified by stroke severity at 3 months (**a**, **b**), 6 months (**c**, **d**), and 1 year (**e**, **f**) after stroke. Abbreviations: mRS, modified Rankin Scale; NIHSS, National Institutes of Health Stroke Scale; SSI, stroke severity index

Of the 3630 patients in the validation cohort, 2188 (60.3 %) were categorized as having mild stroke (NIHSS ≤ 5), 863 (23.8 %) as having moderate stroke (NIHSS > 5 to ≤ 13), and 579 (16.0 %) as having severe stroke (NIHSS > 13). According to the SSI, 2404 (66.2 %) were categorized as having mild stroke (SSI ≤ 5), 630 (17.4 %) as having moderate stroke (SSI > 5 to ≤ 12), and 596 (16.4 %) as having severe stroke (SSI > 12). Figure 2 illustrates the proportions of mortality and good functional outcome (mRS ≤ 2) across the NIHSS and SSI categories at 3 months, 6 months, and 1 year after stroke. With rising stroke severity, as assessed using either the NIHSS or the SSI, the risk of mortality increased and the probability of good functional outcome decreased significantly (all were *P* < 0.001, chi-squared tests for trend).

**Fig. 3:**
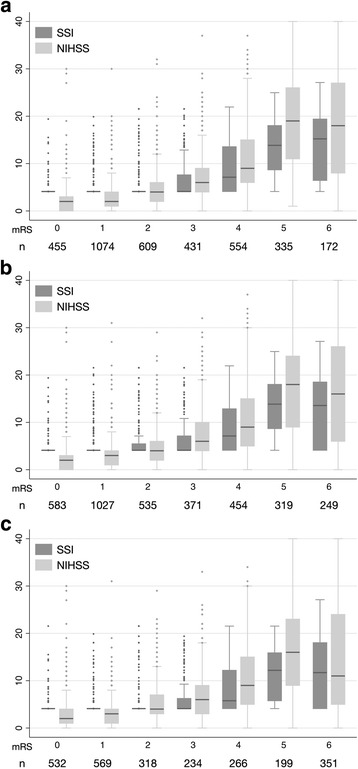
Box-plots showing the distribution of the SSI and the NIHSS across all mRS grades at 3 month (**a**), 6 months (**b**), and 1 year (**c**) after stroke. Abbreviations: mRS, modified Rankin Scale; NIHSS, National Institutes of Health Stroke Scale; SSI, stroke severity index

The SSI correlated significantly with the 3-month mRS (Spearman rho = 0.578; 95 % confidence interval [CI], 0.556–0.600), 6-month mRS (rho = 0.551; 95 % CI, 0.528–0.574), and 1-year mRS (rho = 0.532; 95 % CI 0.504–0.560), indicating a decrease in the magnitude of correlation over time. The admission NIHSS also correlated significantly with the 3-month mRS (rho = 0.674; 95 % CI, 0.656–0.692), 6-month mRS (rho = 0.640; 95 % CI, 0.620–0.659), and 1-year mRS (rho = 0.595; 95 % CI 0.569–0.620). The correlations between the SSI and the mRS were lower than those between the NIHSS and the mRS (*P* < 0.001 for 3-month mRS, *P* < 0.001 for 6-month mRS, and *P* = 0.001 for 1-year mRS). Figure 3 illustrates the distribution of the SSI and the NIHSS across all mRS grades at 3 months, 6 months, and 1 year after stroke. The SSI showed a floor effect with mRS ≤ 2.

Table 3 reports the performance of the logistic regression models for 3-month, 6-month, and 1-year mortality. Based on the Hosmer-Lemeshow statistics, all models demonstrated adequate model fit. For the base models including age, sex, vascular risk factors, and the modified CCI as covariates, the AUCs for 3-month, 6-month, and 1-year mortality models were 0.786, 0.777, and 0.767, respectively. Including the SSI in the models improved model discrimination substantially (AUCs for 3-month, 6-month, and 1-year mortality models, 0.869, 0.844, and 0.823, respectively; all were *P* < 0.001 as compared with the base models). Similarly, model discrimination was enhanced by adding the NIHSS to the base models (AUCs for 3-month, 6-month, and 1-year mortality models, 0.860, 0.837, and 0.816, respectively; all were *P* < 0.001 as compared with the base models). The AUCs of the models including the SSI and models with the NIHSS did not differ significantly (*P* = 0.347 for 3-month mortality, *P* = 0.409 for 6-month mortality, and *P* = 0.353 for 1-year mortality). The IDI also indicated that adding stroke severity, as assessed using either the SSI or the NIHSS, to mortality models significantly improved model discrimination.

**Table 3:** Performance of mortality models for acute ischemic stroke

	AUC (95 % CI)	Hosmer-Lemeshow statistic	*P*	IDI (95 % CI)	*P*
Mortality at 3 months					
Base model	0.786 (0.752–0.819)	6.39	0.604	Reference	
Model with SSI	0.869 (0.844–0.894)	7.07	0.529	0.102 (0.079–0.124)	<0.001
Model with NIHSS	0.860 (0.833–0.887)	7.17	0.519	0.085 (0.064–0.106)	<0.001
Mortality at 6 months					
Base model	0.777 (0.748–0.805)	5.13	0.744	Reference	
Model with SSI	0.844 (0.819–0.869)	5.41	0.713	0.097 (0.077–0.116)	<0.001
Model with NIHSS	0.837 (0.812–0.862)	5.86	0.662	0.082 (0.063–0.100)	<0.001
Mortality at 1 year					
Base model	0.767 (0.741–0.793)	4.30	0.829	Reference	
Model with SSI	0.823 (0.799–0.846)	8.88	0.353	0.101 (0.082–0.121)	<0.001
Model with NIHSS	0.816 (0.793–0.840)	6.42	0.600	0.088 (0.070–0.106)	<0.001

*AUC* area under the receiver operating characteristic curve, *CI* confidence interval, *IDI* integrated discrimination improvement, *NIHSS* National Institutes of Health Stroke Scale, *SSI* stroke severity index

## Discussion

Our study demonstrated that the claims-based SSI could be a feasible proxy indicator for stroke severity when conducting outcomes research using claims data. The predictive validity of the SSI was shown by its significant correlations with the follow-up mRS up to 1 year. The SSI helped predict long-term stroke outcomes, including mortality and functional outcomes, just as the NIHSS did. Adding the SSI to mortality risk models based on claims data for patients with AIS considerably improved model discrimination and the magnitude of improvement was similar to that when the NIHSS was added to the models.

Stroke severity, as assessed using the NIHSS or other stroke scales, is not only a strong predictor of stroke outcomes such as mortality and readmission [27, 28], but also a major determinant of hospital costs for stroke patients [29]. Without information about stroke severity, the results of stroke outcomes research could be biased. A study found that including the NIHSS by linkage with the Get With The Guidelines–stroke registry to a claims-based 30-day mortality model, which was used for profiling hospital performance in AIS treatment, both enhanced model discrimination and improved hospital performance rankings substantially [30]. Nevertheless, the opportunity to link claims data with a nationwide clinical registry is generally unavailable to researchers, and record linkage may be restricted by privacy concerns and regulatory constraints [31]. In circumstances where information on clinical stroke severity is unavailable, the claims-based SSI could be used for risk adjustment in outcomes research using solely claims data.

Various proxy measures of stroke severity have been used in previous claims data-based studies, including length of stay [32], total medical expenditure during stroke hospitalization [33], stroke-related neurological deficits (e.g., hemiplegia and aphasia) and complications (e.g., pneumonia and decubitus ulcers) [33–35], and procedures (e.g., mechanical ventilation and craniotomy) [34, 35]. However, our previous work found that many of these measures might not be valid indicators of stroke severity [25]. On the contrary, the SSI has been externally validated by its strong correlation with clinical stroke severity as measured using the NIHSS [10].

Some may argue that the SSI measure depends on management and treatment given to stroke patients during hospitalization and its performance might thus be affected by variations in practice among physicians and differences in resources across hospitals. Furthermore, physicians may selectively withhold certain management or treatment from some patients, in particular those with very severe stroke because they are expected to die anyway. Despite these concerns, the components of the SSI are widely available in hospitals throughout Taiwan, leading to its consistent performance across cohorts from 4 hospitals of different sizes and types [10]. Because the SSI is superior to other proxy measures of stroke severity [25], for the time being it is a reasonable substitute for a clinical stroke scale in doing research with administrative claims data, in which clinical information is unavailable.

In addition to stroke outcomes research [5, 6, 32, 36], administrative claims data have been widely used in other types of stroke studies, including health care utilization [35, 37, 38], and studies investigating risk factors for stroke [39–41]. However, only a few studies have attempted to use information available in claims data as surrogates for stroke severity [32, 34–37]. With the capability to estimate stroke severity based on claims data, the SSI could be applied in these claims data-based stroke studies. For studies that investigate risk factors for stroke, we could compare the stroke severity between patients with or without the presumed risk factor and, thus, might refine the inferences of such associations. Furthermore, with its ability to predict long-term stroke outcomes, the SSI could assist surveillance of stroke burden using administrative claims data. For example, investigators could examine the temporal trends and spatial variations in patient stroke severity in addition to stroke incidence. The results of such studies may promote effective planning for health care facilities.

Our study has limitations. First, the study patients were enrolled from only 2 hospitals, one being a medical center and the other a regional hospital, and might not be representative of the general population. In Taiwan, approximately 70 % of stroke patients are admitted to medical centers and regional hospitals, with the remainder admitted to district hospitals [35]. However, the seven SSI components are quite ordinary and widely available at each level of hospital providing acute care for patients with stroke. Thus the SSI would not be affected significantly by the size or type of a hospital. Second, distribution of stroke severity is typically skewed toward mild symptoms [42]. As shown in our study, more than half of the patients had mild stroke. However, unlike the NIHSS, the lowest possible value of the SSI was not zero according to the regression equation (Table 1). Consequently, the SSI has a floor effect and is insensitive when discriminating patients with mild stroke. Third, the study results may not be generalized to administrative claims databases other than Taiwan’s NHIRD. Given the kind of administrative information, i.e., administrative billing codes, required, which are typically collected in healthcare systems operating on a fee-for-service basis, the SSI may not be applicable in organizational context that do not have such accounting schemes. More validation studies are required to test the generalizability of this index to other administrative databases and in other healthcare systems. Fourth, the SSI is probably not suitable for case mix adjustment when comparing hospital performance in the future because this method might create an incentive to over-report management and treatment to hospitals that attempt to promote their ranking among hospitals. However, the SSI has its merit in conducting stroke outcome studies with the existing NHIRD datasets [26].

## Conclusions

The claims-based SSI is a valid substitute for the NIHSS for estimating the stroke severity of patients hospitalized for AIS. The SSI correlated with functional outcomes up to 1 year after stroke and it improved case-mix adjustment for mortality to a similar degree as that of the NIHSS. The SSI has the potential to improve stroke research based on administrative claims data.

## Additional file

Additional file 1: Table S1. Predictors of the stroke severity index and their corresponding billing codes in Taiwan’s National Health Insurance fee schedule. **Table S2.** Distribution of the NIHSS score across stroke severity groups stratified by the SSI based on unpublished data from the validation cohorts (*n* = 6617) of our prior study (Sung S-F, et al. J Clin Epidemiol. 2015;68:1292–1300). **Table S3.** Comparison between patients with and without successful linkage. **Table S4**. Comparison between patients with and without follow-up mRS. (PDF 93 kb)

## Abbreviations

AIS: Acute ischemic stroke
AUC: Area under the receiver operating characteristic curve
CCI: Charlson comorbidity index
CI: Confidence interval
ICD-9-CM: International Classification of Diseases, Ninth Revision, Clinical Modification
IDI: Integrated discrimination improvement
mRS: Modified Rankin Scale
NHI: National Health Insurance
NHIRD: National Health Insurance Research Database
NIHSS: National Institutes of Health Stroke Scale
SSI: Stroke severity index
