# Supplementary material for: Validity of a stroke severity index for administrative claims data research: a retrospective cohort study
Source: BMC Health Serv Res. 2016 Sep 22;16:509. doi: 10.1186/s12913-016-1769-8 (PMC5034530; doi:10.1186/s12913-016-1769-8)
Supplement: Additional file 1: Table S1. — Predictors of the stroke severity index and their corresponding billing codes in Taiwan’s National Health Insurance fee schedule. Table S2. Distribution of the NIHSS score across stroke severity groups stratified by the SSI based on unpublished data from the validation cohorts (n = 6617) of our prior study (Sung S-F, et al. J Clin Epidemiol. 2015;68:1292–1300). Table S3. Comparison between patients with and without successful linkage. Table S4. Comparison between patients with and without follow-up mRS. (PDF 93 kb) [file 12913_2016_1769_MOESM1_ESM.pdf]

Additional file 1

Table S1. Predictors of the stroke severity index and their corresponding billing codes in Taiwan's National Health Insurance fee schedule.

Feature	Billing codes
Airway suctioning	47041C, 47042C
Bacterial sensitivity test	13009B, 13009BB, 13010B, 13010BA, 13011B, 13011BB, 13020B
General ward stay	02006K, 02007A, 02008B, 03001K, 03002A, 03003B, 03004B, 03005K, 03006A, 03007B, 03008B, 03026K, 03027A, 03028B, 03029B
ICU stay	02011K, 02012A, 02013B, 03010E, 03010K, 03011A, 03011F, 03012B, 03012G, 03013B, 03013H, 03047E, 03048F, 03049G, 03050H
Nasogastric intubation	47017C, 47018C, 47018CA
Osmotherapy (mannitol or glycerol infusion)	A009633255, A009633266, A009633277, A009745277, A013354277, A015561255, A015561266, A015561277, A016476238, A016476266, A016476277, A031387238, A033425266, A042601238, B014379277, B020322265, B020322277, N012343266, A023733263, A023733265, A023733266, A023733277, A024986209, A024986265, A024986266, A024986277, A025104266, A025104277, A025355266, A025355277, A026793265, A026793266, A026793277, A028475265, A028475277, A029475265, A029475266, A029475277, A034722277, AC23733263, AC23733266, AC23733277, AC24986265, AC24986266, AC24986277, AC28475277, AC29475265, B006604277, B017082263, B017082277, B017728265, B017728277
Urinary catheterization	47013C, 47014C

ICU intensive care unit.

Table S2. Distribution of the NIHSS score across stroke severity groups stratified by the SSI based on unpublished data from the validation cohorts (n = 6617) of our prior study (Sung S-F, et al. *J Clin Epidemiol*. 2015;68:1292–1300).

	Mild (SSI ≤ 5)	Moderate (SSI > 5 to ≤ 12)	Severe (SSI > 12)
NIHSS, mean (SD)	3.9 (3.6)	9.0 (7.1)	18.4 (9.3)
NIHSS, median (IQR)	3 (1–5)	7 (4–12)	19 (11–26)

Based on linear regression analysis, the NIHSS score can be predicted as follows: NIHSS = 1.1722×SSI−0.7533 ($P<0.0001$).

IQR interquartile range, *NIHSS* National Institutes of Health Stroke Scale, *SD* standard deviation, *SSI* stroke severity index.

Table S3. Comparison between patients with and without successful linkage.

		Successful linkage		
	Total (n = 5586)	Yes (n = 4778)	No (n = 808)	*P*
Age, mean (SD)	67.5 (12.6)	67.4 (12.7)	68.5 (11.9)	0.022
Female, n (%)	2241 (40.1)	1909 (40.0)	332 (41.1)	0.630
NIHSS, mean (SD)	7.4 (8.2)	7.3 (8.0)	8.4 (9.1)	< 0.001
NIHSS group				0.075
≤ 5	3308 (59.2)	2854 (59.7)	454 (56.2)	
> 5 to ≤ 13	1281 (22.9)	1092 (22.9)	189 (23.4)	
> 13	997 (17.9)	832 (17.4)	165 (20.4)	

NIHSS National Institutes of Health Stroke Scale, *SD* standard deviation.

Table S4. Comparison between patients with and without follow-up mRS.

		Available mRS at 3 months		
	Total (n = 4778)	Yes (n = 3630)	No (n = 1148)	*P*
Age, mean (SD)	67.4 (12.7)	67.6 (12.7)	66.7 (12.7)	0.031
Female, n (%)	1909 (40.0)	1474 (40.6)	435 (37.9)	0.102
NIHSS, mean (SD)	7.3 (8.0)	7.1 (7.8)	7.9 (8.7)	0.002
NIHSS group				< 0.001
≤ 5	2854 (59.7)	2188 (60.3)	666 (58.0)	
> 5 to ≤ 13	1092 (22.9)	863 (23.8)	229 (20.0)	
> 13	832 (17.4)	579 (16.0)	253 (22.0)	
SSI, mean (SD)	7.0 (5.1)	6.8 (4.8)	7.8 (5.8)	< 0.001
Pearson correlation between NIHSS and SSI (95% CI)	0.712 (0.697-0.726)	0.699 (0.682-0.715)	0.743 (0.715-0.768)	

CI confidence interval, *mRS* modified Rankin Scale*, NIHSS* National Institutes of Health Stroke Scale, *SD* standard deviation, *SSI* stroke severity index.
